# Buttermilk ice cream—New method for buttermilk utilization

**DOI:** 10.1002/fsn3.1429

**Published:** 2020-02-14

**Authors:** Katarzyna Szkolnicka, Izabela Dmytrów, Anna Mituniewicz‐Małek

**Affiliations:** ^1^ Department of Toxicology, Dairy Technology and Food Storage Faculty of Food Sciences and Fisheries West Pomeranian University of Technology Szczecin Poland

**Keywords:** buttermilk, color, ice cream, sensory assessment, texture

## Abstract

Buttermilk, the by‐product of butter production, due to good technological features and excellent nutritional and health‐promoting properties finds more and more applications in food industry. Considerable amount of polar lipids causes that buttermilk exhibits emulsifying and stabilizing effect and may be used to improve the product quality. The study aimed to design new kind of ice cream, in which all milk is substituted by buttermilk. Within the study, we compared physicochemical parameters, color, texture, and sensory properties of control milk ice cream (C), ice cream from sweet buttermilk (SB), and ice cream from cultured buttermilk (CB). Ice cream was tested on the production day, and some characteristics were tested also after 14 and 28 days of storage at −18 ± 1°C. The study showed that samples of ice cream from cultured buttermilk had the highest acidity and were the most resistant to melting. The samples did not differ in over‐run value. The use of buttermilk influenced the texture of ice cream and product from sweet buttermilk had the highest stickiness during the storage. The color analysis showed that the highest lightness parameter had ice cream from cultured buttermilk, while samples from sweet buttermilk had the most greenish‐yellow characteristics. All the obtained products had good sensory characteristics, only cultured buttermilk ice cream slightly deteriorated after 28 storage days. Good quality properties cause that buttermilk may be successfully used as substitution of milk in ice‐cream formula and may improve its quality by exhibiting of some emulsifying stabilizing effect.

## Graphical abstract

The study aimed to develop a new kind of ice cream in which all milk is replaced with buttermilk. This by‐product of butter churning is characterized with similar to skimmed milk composition and excellent nutritional value. Emulsifying properties of buttermilk make that it may be used as emulsifier and improve the quality of ice cream. Obtained buttermilk ice cream had good quality properties, thus buttermilk may be successfully used in ice‐cream production.

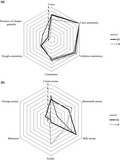

## 1 INTRODUCTION

Buttermilk is a by‐product released during churning of cream in the process of butter making. It is a remaining liquid phase containing water and water‐soluble components of cream. An overall composition of buttermilk is very similar to skimmed milk, and buttermilk contains lactose, proteins (casein and serum proteins), and minerals (De Bassi et al., 2011; Gebreselassie, Abrahamsen, Beyene, Abay, & Narvhus, 2016). According to Eurostat (2019), over 29% of milk processed in European Union is used for butter production and the amount of butter obtained in 2017 was 2.4 million tonnes. Since the yield of butter production is about 50% (Morin, Jiménez‐Flores, & Pouliot, 2007), the buttermilk production amounted similar value. Depending on the acidity, there are distinguished two types of buttermilk, that is sweet buttermilk made from sweet cream and cultured (fermented) buttermilk made either from cultured cream or by fermentation of sweet buttermilk by starter culture of mesophilic lactic acid bacteria (Gebreselassie et al., 2016).

Buttermilk has attracted particular interest of food technologists due to considerable content of milk fat globule membrane (MFGM) constituents. This biological membrane, composed from polar lipids and proteins, coats fat droplets in milk and cream and ensures their stability and integrity. During churning of cream, MFGM is destroyed and its components are released into buttermilk where they determinate beneficial health‐related properties of this product, thus buttermilk can play an important role in human nutrition (Conway, Couture, Gauthier, Pouliot, & Lamarche, 2014; Vanderghem et al., 2010). Polar lipids of MFGM are phospholipids and sphingolipids. The concentration of those components in buttermilk is five times higher than in whole milk. It is established that phospholipids have cholesterol lowering and anti‐inflammatory effects and positively impact the functions of the nervous system (El‐Loly, 2011; Jiménez‐Flores & Brisson, 2008; Liutkevičius et al., 2016). About 19% of buttermilk proteins are proteins originated from MFGM (Britten, Lamothe, & Robitaille, 2008). Natural role of this proteins is to improve the defense system of new‐born, thus they are shown to have antioxidant and antimicrobial properties (Conway, Gauthier, & Pouliot, 2012; Dewettinck et al., 2008; Mills, Ross, Hill, Fitzgerald, & Stanton, 2011). What is especially important in technological point of view, both polar lipids and MFGM proteins have emulsifying properties (Liutkevičius et al., 2016; Vanderghem et al., 2010) what causes that buttermilk may play the role of natural emulsifier.

Due to good nutritional characteristics, low acquisition costs, similarity to skimmed milk, good sensory features, emulsifying properties as well as positive influence on human health, buttermilk is suitable material to develop new food products with functional properties (Vanderghem et al., 2010). Buttermilk finds applications mainly in dairy technology. It may be used as a raw material for the production of fermented dairy type beverages (De Bassi et al., 2011; Liutkevičius et al., 2016) or fermented drinks with juice addition (Mudgil, Barak, & Darji, 2016). Buttermilk fermentation may be performed by probiotic bacteria (Burns et al., 2010; Burns, Vinderola, Molinari, & Reinheimer, 2008). Due to water holding capacity, buttermilk powder improves structure of yogurt preventing syneresis (Romeih, Abdel‐Hamid, & Awad, 2014) and may be used in low‐fat yogurt production (Zhao, Feng, Ren, & Mao, 2018). Due to the ability to improve texture, viscosity, moisture retention, and flavor, buttermilk is used in production of reduced‐fat cheese (Mistry, 2001) and pizza cheese (Govindasamy‐Lucey et al., 2007). Beside dairy industry, buttermilk finds applications in production of bread (Madenci & Bilgiçli, 2014), microencapsulation of omega‐3 oils (Augustin et al., 2015), and fruit‐flavored beverages (Shaikh & Rathi, 2009).

According to Marshall (2003) and Goff (2003), buttermilk may be favorable component of ice‐cream mix, which improves the texture of ice cream without emulsifiers addition. The authors also suggested that buttermilk has beneficial effect on the whippability of mixes, thereby may increase over‐run of ice cream. However we have not found the literature concerning the effect of buttermilk addition on ice‐cream quality.

For that reason, the study aimed to determine the quality characteristics of ice cream made of sweet and cultured buttermilk during 4‐week storage period.

## 2 MATERIAL AND METHODS

### 2.1 Materials

Pasteurized skimmed milk (0.5% fat), pasteurized cream (30% fat), and sucrose were purchased at a local marked. Skimmed buttermilk powder containing 0.5% fat and 95% total solids was supplied by Spomlek Company (Poland) and skimmed milk powder (0.2% fat, 96% total solids) by SM Gostyń (Poland). Freeze dried mesophilic lactic acid bacteria starter culture consisting *Lactococcus lactis* ssp *lactis* and *Lactococcus lactis* ssp *cremoris* (MO 242) was produced by SACCO Srl (Italy). All the chemical reagents used were analytical grade.

### 2.2 Buttermilk production

Buttermilk was manufactured by churning nonhomogenized pasteurized cream (30% fat) in laboratory scale churning machine. To ensure high microbiological quality, directly after production buttermilk underwent pasteurization process (72°C, 10 min). Obtained buttermilk was divided into two batches. One of them constituted sweet buttermilk. The other one was inoculated with 0.6 g/L of mesophilic lactic acid bacteria starter culture, incubated overnight at 25 ± 1°C and cultured buttermilk was obtained.

### 2.3 Ice‐Cream production

The production of ice cream was conducted in laboratory conditions. Ice‐cream mixes were frozen in ice‐cream machine with 1.5 L capacity equipped with self‐cooling compressor freezing to approximately −35°C (Springlane GmbH, Germany). Within the study, three kinds of ice cream were prepared: (a) control ice cream (C) made from skimmed milk, cream, skimmed milk powder and sucrose; (b) ice cream from sweet buttermilk (SB) made from sweet buttermilk, cream, buttermilk powder, and sucrose; (c) ice cream from cultured buttermilk (CB) made from cultured buttermilk, cream, buttermilk powder, and sucrose. Liquid components for ice‐cream productions had the temperature of 5 ± 1°C. Because we focused to observe the positive impact of buttermilk on ice‐cream structure, we did not applied stabilizers and emulsifiers to the mixes. The ingredients were combined in calculated proportions to obtain 10% fat, 12% MSNF (milk solids‐not‐fat), and 12% sucrose in the mixture. The ice‐cream mixes did not underwent the pasteurization process because all three treatments were processed in the same manner. Product with cultured in order to retain alive lactic acid bacteria could not be thermally treated, thus all the products were not. However, used ingredients were previously pasteurized what should ensure microbiological safety. The mixtures were homogenized with home‐type blender and aged for 2 hr at 4 ± 1°C. Short aging time was dictated by the lack of stabilizers and emulsifiers in the mix. Then, the mixtures were simultaneously frozen and mixed in ice‐cream machine. The freezing time was 1 hr. Directly after production, the temperature of ice cream was −10°C. Then, ice‐cream samples were filled into plastic‐covered containers and hardened to the temperature of −18°C. The samples of ice cream were tested on the production day and after 2 and 4 weeks of storage at −18 ± 1°C. Short storage period was dictated by the lack of emulsifiers and stabilizers in ice‐cream mixtures, thus the products are not designed for long storage.

### 2.4 Methods

#### 2.4.1 Raw material analyses

Protein, fat and total solids content and titratable acidity of skimmed milk and buttermilk were analyzed in accordance with Official Analytical Methods (AOAC, 1997). Protein content was determined by Kjeldahl method and fat content by the Gerber method. Total solids were determined by drying at 105°C. Titratable acidity, expressed as a percentage of lactic acid, was assessed by titration using 0.25 N NaOH solution. The pH was evaluated with a pH meter (model HI98128, HANNA Instruments).

#### 2.4.2 Ice‐cream analyses

On the production day, following analyses were performed: protein, fat and total solids content, titratable acidity, pH, over‐run, meltdown rate, texture analysis, color, and sensory assessment. After 2‐ and 4‐week storage period, total solids content, titratable acidity, pH, and texture parameters were tested and sensory analysis was performed.

Total solids content and titratable acidity presented as % lactic acid were evaluated in melted samples (AOAC, 1997) as well as pH measured with the same pH meter.

Over‐run of ice cream is an indicator informing how much air was incorporated into ice‐cream mix during freezing. Over‐run of the ice cream was assessment as a comparison of the weight of equal volume of ice‐cream mix and ice cream. The value of over‐run was calculated according to following formula (Akin et al., 2007):

$$\text{Over}\;-\;\text{run}\;\left[\%\right]\;=\;\frac{\text{weight}\;\text{of}\;\text{ice}\;-\;\text{cream}\;\text{mix}\;-\;\text{weight}\;\text{of}\;\text{ice}\;\text{cream}}{\text{weight}\;\text{of}\;\text{ice}\;\text{cream}}\;\times \;100.$$

Meltdown rate [%] was determined in accordance with the method presented by Milani and Koocheki (2011) with some modifications. 25 g scoop of ice cream was placed on a plastic strainer with openings of 1 × 1 mm, located on top of a beaker. After 60 min at 20 ± 0.1°C, the weight of the melted sample collected in the beaker was measured. Meltdown rate was expressed as percent of the initial weight of ice‐cream scoop:

$$\text{Meltdown}\;\text{rate}\;\left[\%\right]\;=\;\frac{\text{weight}\;\text{of}\;\text{melted}\;\text{sample}}{\text{weight}\;\text{of}\;\text{scoop}}\;\times \;100.$$

The study included also texture profile analysis (TPA) which was performed using texture analyzer TA.XT plus (Stable Micro System). The test included the measurement of hardness (the peak force during the penetration of the sample) and stickiness (the negative peak force during the withdrawal of the probe). The samples were penetrated with 10‐mm diameter cylindrical aluminum probe with the trigger force 5 g, penetration distance 25 mm, and test speed 5 m/s. In order to simulate the conditions during ice‐cream consumption, the samples were tempered at −6°C for 1 hr and assessments were carried out at ambient temperature (20 ± 1 ºC).

The color parameters L*, a*, and b* were determined using the CIELAB system on the surface of ice‐cream samples with portable colorimeter (model FRU® WR‐18, Shenzhen Wave Optoelectronics Technology Co., Ltd) using 8 mm aperture. Before test, colorimeter was calibrated with standard white plate.

Sensory evaluation was performed in accordance with international guidelines (PN‐ISO 6658:^1998^; PN‐ISO 22935‐2:^2013^‐07) by a panel of 6 evaluators trained in dairy product assessment. The evaluation included assessment of overall sensory quality and sensory profile analysis. The overall sensory quality was estimated by grading the sensory attributes in the range from 1 (very poor quality) to five points (very good quality). The mean scores for each attribute were used to calculate overall sensory quality which was determined as weighted average of the scores. The weights reflected the influence of each attribute on determination of overall sensory quality and were as follow: consistency—0.45; taste—0.2; flavor—0.15; color—0.1; and appearance—0.1. In sensory profile analysis, the evaluators were asked to rate the sensory attributes of ice cream using 9‐point intensity scale with 0—lack of attribute, 1—the lowest intensity, and 9—the highest intensity. The sensory attributes were as follows: color, color uniformity, uniform consistency, creaminess, rough consistency, presence of clumps/granules, cream aroma, buttermilk aroma, milk aroma, acidity, bitterness, and foreign aroma (Table 1). The ice‐cream samples were coded using a three‐digit random numbers and were served at room temperature. The evaluators had separate stands, and each of them received water to clean palate.

**Table 1:** Attributes of buttermilk ice‐cream sensory analyses

Attribute	Definition	Scale
Assessment of overall sensory quality (1–5)		
Consistency	The liking of consistency	1 ‐ extremely dislike, 5 ‐ extremely like
Taste	The liking of taste	1 ‐ extremely dislike, 5 ‐ extremely like
Flavor	The liking of flavor	1 ‐ extremely dislike, 5 ‐ extremely like
Color	The liking of color	1 ‐ extremely dislike, 5 ‐ extremely like
Appearance	The liking of appearance	1 ‐ extremely dislike, 5 ‐ extremely like
Sensory profile analysis (0–9)		
Color	White to dark ivory color	0 ‐ white, 9 ‐ dark ivory
Color uniformity	Uniform color in all sample	0 ‐ totally ununiform, 9 ‐ totally uniform
Uniform consistency	Uniform and homogenous consistency	0 ‐ totally ununiform, 9 ‐ totally uniform
Creaminess	Smooth and creamy, easy to scoop consistency	0 ‐ lack of attribute, 9 ‐ intensive attribute
Rough consistency	Ice crystals perception, sandiness	0 ‐ lack of attribute, 9 ‐ intensive attribute
Presence of clumps/granules	Presence of particles detectable in mouth	0 ‐ lack of attribute, 9 ‐ intensive attribute
Cream aroma	Aroma associated with creamy products	0 ‐ lack of attribute, 9 ‐ intensive attribute
Buttermilk aroma	Aroma associated with buttermilk	0 ‐ lack of attribute, 9 ‐ intensive attribute
Milk aroma	Aroma associated with milk	0 ‐ lack of attribute, 9 ‐ intensive attribute
Acidity	Taste sensation typical for products with lactic acid	0 ‐ lack of attribute, 9 ‐ intensive attribute
Bitterness	Sharp taste typical for caffeine or quinine	0 ‐ lack of attribute, 9 ‐ intensive attribute
Foreign aroma	Presence of off‐flavors typically not present in ice cream	0 ‐ lack of attribute, 9 ‐ intensive attribute

### 2.5 Statistical analysis

The analyses were performed in triplicate excluding texture and color measurements which were performed with five repetitions. Obtained results were statistically analyzed at the significance level *p* < .05 using Statistica 13.1 Software (StatSoft Inc.). Mean values and standard deviations were calculated, then the values of the quality properties were compared by Tukey's HSD test. The influence of the composition on analyzed ice‐cream properties was evaluated by ANOVA test.

## 3 RESULTS AND DISCUSSION

### 3.1 Composition and acidity of raw materials and ice cream

Sweet buttermilk obtained during churning of pasteurized cream (30% fat) had composition very similar to skimmed milk used as main raw material for control ice‐cream production. They were significantly different (*p* < .05) only in case of fat content, which was 0.8% and 0.5%, respectively, in buttermilk and milk. They did not differ in protein content (respectively 3.3% and 3.4%), total solids (10.0% and 9.9%), pH (6.75 and 6.66), and titratable acidity (0.138% and 0.145% of lactic acid). The content of fat, protein, and total solids in the buttermilk was higher than in buttermilk tested by Gassi, Famelart, and Lopez (2008) which contained up to 0.54% fat, 2.94% protein, and 9.16% total solids. However, de Bassi et al. (2011) obtained buttermilk with higher nutrients content (1.18% fat, 4.44% protein, and 12.61% total solids). Both cited authors, analogous to our research, obtained buttermilk from sweet cream. According to Sodini, Morin, Olabi, and Jiménez‐Flores (2006), the use of ether sweet or sour cream for butter production influences the buttermilk composition. Gassi et al. (2008) pointed out that buttermilk properties depend on origin of milk, heat treatment of cream, and butter technology (slow or rapid churning).

After fermentation with mesophilic lactic acid bacteria, obtained cultured buttermilk had significantly (*p* < .05) higher acidity (pH 4.21, 0.795% of lactic acid). The differences in fat content in milk and buttermilk transferred into significantly (*p* < .05) lower fat content in control ice cream (9,6%) than in ice cream made from sweet and cultured buttermilk (ice cream SB ‐ 9.8%, ice cream CB 9.9%). Moreover, control ice cream was characterized with significantly (*p* < .05) higher protein content (4.8%) than buttermilk ice creams (SB ‐ 4.5%, CB ‐ 4.6%). Fat and protein content were measured only on a day of ice‐cream production. pH, titratable acidity, and total solids content were analyzed also after 2 and 4 weeks of storage, and the results are presented in Table 2.

**Table 2:** pH, titratable acidity, and total solids content during the storage of control and buttermilk ice cream

Day	Product		
	C	SB	CB
pH			
1	6.45 ± 0.01 ^a,B^	6.53 ± 0.01 ^c,C^	5.16 ± 0.01 ^a,A^
14	6.53 ± 0.01 ^b,C^	6.33 ± 0.01 ^a,B^	5.46 ± 0.01 ^b,A^
28	6.59 ± 0.01 ^c,C^	6.40 ± 0.01 ^b,B^	5.66 ± 0.01 ^c,A^
% Lactic acid			
1	0.192 ± 0.005 ^a,A^	0.183 ± 0.005 ^a,A^	0.558 ± 0.000 ^a,B^
14	0.184 ± 0.004 ^a,A^	0.232 ± 0.007 ^b,B^	0.585 ± 0.005 ^b,C^
28	0.183 ± 0.002 ^a,A^	0.238 ± 0.005 ^b,B^	0.583 ± 0.003 ^c,C^
Total solids, %			
1	34.26 ± 0.14 ^a,A^	34.53 ± 0.52 ^a,A^	34.81 ± 1.68 ^a,A^
14	33.55 ± 1.01 ^a,A^	36.32 ± 2.63 ^a,A^	34.71 ± 2.11 ^a,A^
28	32.69 ± 1.96 ^a,B^	37.47 ± 0.76 ^a,A^	37.74 ± 1.98 ^a,A^

Note

Different letters in superscript indicate statistically significant (*p* < .05) differences between mean values in columns (lowercase letters) and in rows (uppercase letters).

Abbreviations: CB, ice cream from cultured buttermilk; C, control; SB, ice cream from sweet buttermilk.

The differences in pH acidity of obtained ice cream were statistically significant (*p* < .05). On the production day, the highest pH value had ice cream SB from sweet buttermilk. After two and four weeks of storage, the highest pH had control. As a result of low pH of cultured buttermilk, ice cream CB was characterized with the lowest pH value throughout all storage period. pH of ice cream from cultured buttermilk and from milk (control) increased during storage period. In case of SB product, pH decreased during first two weeks and then also increased (Table 2). Titratable acidity expressed as lactic acid content was also tested (Table 2). Analogically to pH, the highest lactic acid content during all study had ice cream from cultured buttermilk (CB) and the lowest acidity had product SB (after 1 day) and control (after 14 and 28 days of storage). Titratable acidity of control was stable during storage time, while in case of products from buttermilk, the value of this parameter gradually increased (Table 2). Total solid content did not change significantly (*p* < .05) during storage period and did not differ between the samples. Only after 4‐week storage, control sample had significantly (*p* < .05) lower total solid content than both kinds of buttermilk ice cream (Table 2).

### 3.2 Over‐run and melting properties

The incorporation of air into the ice‐cream mix which takes place during freezing, causes that the volume of final product increases. This increase is referred as over‐run and is one of the most important characteristics of frozen desserts (Marshall, 2003). The volume of incorporated air affects the sensory properties as well as the price of ice cream. It also provides a light texture of ice cream and has influence on melting characteristics (Rezaei, Khomeiri, Aalami, & Kashaninejad, 2014). Over‐run and melting properties were analyzed on a production day, and the results are presented in Table 2.

Over‐run values of tested ice creams were in the range 47.3%–55.5%. Buttermilk ice creams had higher over‐run than control ice cream; however, the differences were statistically insignificant (*p* > .05) (Table 3). It is important to mention that ice‐cream machine in which production was performed did not allowed to control the air admission into ice‐cream mixes and the over‐run value resulted from ice‐cream mixes properties. Our results are in line with the work of Marshall and Arbuckle (1996) who pointed out that over‐run of ice cream should range from 30% to 60%. Over‐run values obtained by other authors are differentiated. Ice cream with milk protein concentrates manufactured by Alvarez, Wolters, Vodovotz, and Ji (2005) had higher over‐run ranging 65.04%–74.01%. In turn, ice cream with different stabilizers produced by BahramParvar, Haddad Khodaparast, and Ravazi (2009) had much lower over‐run (18.8%–28.6%). Lower over‐run values were found also by Akalin, Karagözlü, and Ünal (2008) in reduced‐fat and low‐fat ice cream (25.3%–39.2%). Muse and Hartel (2004) in ice cream with different sweeteners and emulsifiers levels obtained similar over‐run value to our study (37.7%–71.3%). The diversification of over‐run values points out that this characteristic is influenced by ice‐cream composition as well as method of production.

**Table 3:** Over‐run and melting rate of control and buttermilk ice cream

Product	Over‐run, %	Melting rate, %
C	47.3 ± 5.1 ^a^	54.7 ± 1.0 ^b^
SB	55.5 ± 4.1 ^a^	65.9 ± 1.1 ^c^
CB	53.7 ± 4.9 ^a^	42.6 ± 2.9 ^a^
One‐factor ANOVA p‐value	0.086824	0.000000*

Note

Different letters in superscript indicate statistically significant (*p* < .05) differences between mean values in columns.

Abbreviations: CB, ice cream from cultured buttermilk; C, control; SB, ice cream from sweet buttermilk.

* Indicates statistically significant influence of kind of product on analyzed indicator.

Resistance to melting is one of the pivotal factors determining ice‐cream quality. The mean values of melting rate that is the percentage of ice‐cream scoop which get melted during one hour at 20°C are presented in Table 3. It was found that melting rates were significantly different (*p* < .05). The most susceptible to melting was sample from sweet buttermilk, and the most resistant was sample from cultured buttermilk. According to Goff (2003), emulsifiers improve melting resistance of ice cream, thus we may conclude that only cultured buttermilk achieving this effect. In order to facilitate the comparison of our results with results of other authors, the data may be presented as a percentage of the initial mass of ice‐cream scoop which get melted each minute (melting rate results were divided by 60). In this form, our results are 0.91% × min^−1^ for control; 1.10%×min^−1^ for ice cream from sweet buttermilk; and 0.71% × min^‐1^ for ice cream from cultured buttermilk; however, those are mean values and melting process could change over time. Those values are close to results obtained by Bolliger, Goff, and Tharp (2000) which for ice cream with emulsifiers addition were 0.8%–1.0% × min^−1^. Ice cream with milk protein concentrates tested by Alvarez et al. (2005) was more resistant for melting and melting rate was 0.26%–0.29% × min^−1^.

### 3.3 Hardness and Stickiness

The texture of frozen dairy products results from their composition, as well as processing conditions (BahramParvar, MazaheriTehrani, & Razavin, 2013; El‐Nagar, Clowes, Tudorică, Kuri, & Brennan, 2002). The values of the main textural attributes, that is hardness and stickiness, during the storage of buttermilk ice cream are shown in Table 4.

**Table 4:** Hardness and stickiness of control and buttermilk ice cream during the storage

Day	Product		
	C	SB	CB
Hardness, *N*			
1	0.563 ± 0.079 ^a,B^	0.361 ± 0.021 ^a,A^	0.574 ± 0.045 ^a,B^
14	2.320 ± 0.845 ^b,B^	1.973 ± 0.401 ^b,A,B^	1.432 ± 0.289 ^b,A^
28	1.646 ± 0.148 ^b,A^	1.733 ± 0.266 ^b,A,B^	2.335 ± 0.222 ^c,B^
Stickiness			
1	−84.623 ± 17.928 ^a,B^	−50.005 ± 8.847 ^a,A^	−93.786 ± 9.172 ^b,B^
14	−81.029 ± 38.977 ^a,A^	−209.001 ± 49.727 ^c,B^	−66.737 ± 16.257 ^a,A^
28	−51.4715 ± 6.222 ^a,A^	−118.615 ± 12.668 ^b,B^	−58.156 ± 18.291 ^a,A^

Note

Different letters in superscript indicate statistically significant (*p* < .05) differences between mean values in columns (lowercase letters) and in rows (uppercase letters).

Abbreviations: CB, ice cream from cultured buttermilk; C, control; SB, ice cream from sweet buttermilk.

Hardness of analyzed ice cream was varied; however, in all samples, the increase of this parameter was observed. On the day of production, ice cream from sweet buttermilk had the lowest hardness (Table 4). The influence of ice phase volume on the increase of hardness of ice cream was stated by Muse and Hartel (2004). Before first analysis, samples were hardened at −18°C only for 1 hr and may be referred as soft‐serve product. During longer freezing, hardening process has been completed and we obtained hard ice cream. Beside ice phase volume, hardness of ice cream is negatively affected by fat content (BahramParvar et al., 2013) and over‐run (Muse & Hartel, 2004).

Stickiness of ice cream was also varied. In control product, it did not change during a time. In product SB, we observed the increase of stickiness. In contrary, the stickiness of ice cream from cultured buttermilk decreased. On the first day, sweet buttermilk ice cream had the lowest and on 14th and 21st day the highest stickiness. Ice cream from cultured buttermilk did not differ from control during all study period (Table 3). In the study, we wanted to investigate, if buttermilk, due to polar lipid presence, may act as stabilizer of ice cream. Soukoulis, Chandrinos, and Tzia (2008), Milani and Koocheki (2011), and BahramParvar et al. (2013) stated that stabilizers decrease hardness and increase stickiness of ice cream. This effect is connected with the prevention of ice recrystallization and is observed during the product storage. The use of sweet buttermilk distinctly increased the stickiness during the storage in comparison with control ice cream; however, the hardness of control ice cream and sweet buttermilk ice cream (SB) did not differ significantly on 14th and 28th day (Table 3). This results allow us to state that sweet buttermilk has some stabilizing effect by stickiness increasing, although cultured buttermilk does not stabilize the texture of ice cream.

### 3.4 Color

**Figure 1:**
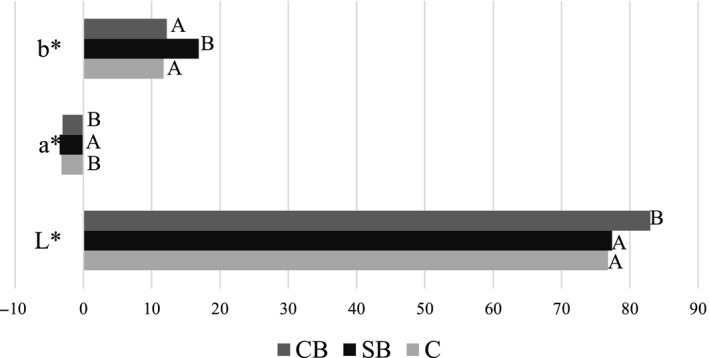
Color parameters (L*, a*, b*) of control and buttermilk ice cream. Different letters in bars of the same coordinate indicate statistically significant differences (*p* < .05). CB, ice cream from cultured buttermilk; C, control; SB, ice cream from sweet buttermilk

Color of the product is strongly connected with consumers’ acceptance and is important also in the case of dairy products like ice cream. However, the literature concerning this aspect is very limited. Figure 1 presents color parameters measured on the production day of tested ice cream.

The color of obtained samples, due to lack of flavor additives, should be characteristic for typical ice cream, that is light ivory and homogenous. The analysis performed with the use of colorimeter showed that obtained products had greenish‐yellowish color characteristics what is indicated by negative values of a* coordinate (greenish) and positive values of b* coordinate (yellowish). Product SB from sweet buttermilk had significantly higher absolute values of a* and b*, thus had more intense greenish‐yellowish color (Figure 1). García‐Pérez et al. (2005) and Cais‐Sokolińska and Pikul (2006) in the study on fermented milk color parameters stated that the lightness index of fermented milk products is determined by their acidity and pH declination is correlated with lightness decrease. Those finding are in contrary to our results, which showed that ice cream CB from cultured buttermilk, which had the highest acidity, had the highest L* parameter (Figure 1). Different findings about the influence of pH on the lightness in our study may point that the state of matter (we investigated solid and quoted authors tested liquid samples) may contribute to the color change. Beside small differences, color parameters of buttermilk ice creams were very similar to control and typical for ice cream. The range of lightness value obtained in our study (76.78 – 83.01) was lower than results of Roland et al. who obtained ice cream with L* value 84–89 (Roland, Phillips, & Boor, 1999a) and 90–95 (Roland, Phillips, & Boor, 1999b). They stated that lightness is influenced by fat content and fat replacers addition to ice cream. Much lower value of L* (60–67) was stated by Sagdic, Ozturk, Cankurt, and Tornuk (2012) who examined probiotic ice cream with phenolic compounds addition. The L* values similar to our findings were stated by Akesowan (2009) (78.96–85.69) who concluded that addition of soy protein isolate reduces ice‐cream lightness. The a* and b* values of our study (respectively from −3.06 to −3.47 and from 11.73 to 16.86) were similar to values of Akesowan (2009) (a* from −1.2 to −4.2 and b* from 15.5 to 16.9) but differ from results of Roland et al. (1999a) (a* from −0.6 to 0.4 and b* from 4.1 to 5.4) and Roland et al. (1999b) (a* from −0.7 to 0.8 and b* from 5.6 to 7.8).

### 3.5 Sensory analysis

**Figure 2:**
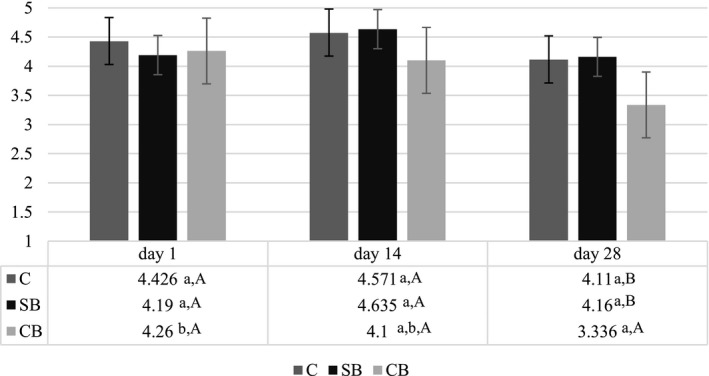
Overall sensory quality of control and buttermilk ice cream. Different letters in superscript indicate statistically significant (*p* < .05) differences between mean values in columns (lowercase letters) and in rows (uppercase letters). CB, ice cream from cultured buttermilk; C, control; SB, ice cream from sweet buttermilk

**Figure 3:**
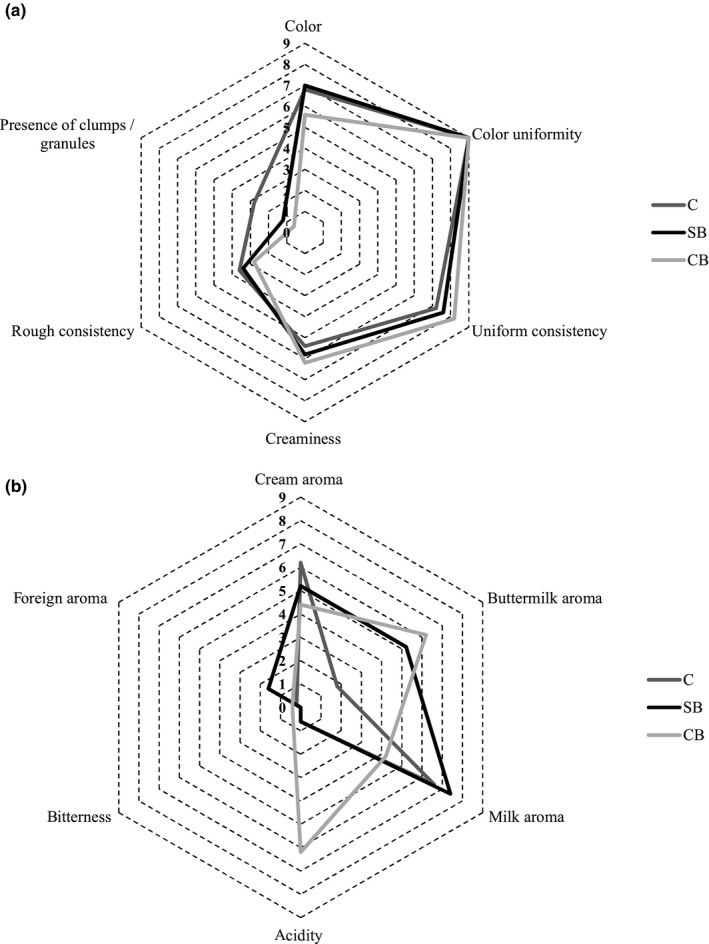
Sensory profile analysis of control and buttermilk ice cream on the production day. A, color and consistency; B, taste and flavor; CB, ice cream from cultured buttermilk; C, control; SB, ice cream from sweet buttermilk

During the storage of buttermilk ice cream, the overall sensory quality was assessed (Figure 2). In addition, on the production day, sensory profile analysis was performed (Figure 3).

The most important sensory characteristics of ice cream, which determine consumers’ acceptance, are consistency (creaminess, ice crystals perception), taste and mouthfeel, flavor, color, and appearance (Stampanoni, Piccinali, & Sigrist, 1996). Based on these attributes, we have determined the overall sensory quality, which was presented in 1–5 point hedonic scale. All the analyzed samples did not differ (*p* < .05) in overall sensory quality on 1st and 14th day of study. On 28th day, the quality of ice cream CB from cultured buttermilk was significantly worse (*p* < .05) because of its more coarse and icy consistency. Only in this product, statistically significant (*p* < .05) deterioration between 1st and 28th day was observed. Control ice cream and ice cream from sweet buttermilk did not change significantly over the storage (*p* < .05).

Sensory profile analysis performed on the production day revealed that among all analyzed attributes, the samples differed only in acidity. The other differences found by evaluators, mainly in taste and flavor attributes (Figure 3b), were statistically insignificant (*p* < .05). Consistency and color (Figure 3a) of tested products were typical for ice cream. Evaluators found some differences in presence of clumps/granules. In case of this parameter, 0 value means lack of particles detectable in mouth. Control ice cream had higher score (2.8) in comparison to ice cream from sweet and cultured buttermilk (1.2 and 0.6, respectively); however, this difference was statistically insignificant. The presence of detectable particles may be caused by the lack of emulsifiers in the mix, not enough homogenization or production of ice cream by home‐type method. In this context, buttermilk seems to be better raw material than milk. Color of ice cream CB from cultured buttermilk was assessed as more white what is in line with measurement with colorimeter (Figure 1); however, in case of sensory profile analysis, this difference was not statistically significant (Figure 3a). It is stated that addition of emulsifiers results in smoother body and higher creaminess of ice cream (Goff, 2003). Based on the results of sensory analysis, we may conclude that substitution of milk with sweet buttermilk leads to product with similar sensory quality that ice cream from milk. Cultured buttermilk is less suitable for hardened ice‐cream production, but may be used for soft ice cream served directly after production process. The literature also indicate good sensory quality of dairy products made from buttermilk. According to Marshall (2003), the addition of buttermilk to ice‐cream formulation contributes to richness of ice‐cream flavor. De Bassi et al. (2011) and Liutkevičius et al. (2016) concluded that fermented beverages from buttermilk have acceptable to consumers sensory properties. In the work of Zhao et al. (2018) on low‐fat yogurt with buttermilk, it is stated that buttermilk positively influence sensory properties by decrease of sourness, increase of milky flavor, and improvement of texture.

## 4 CONCLUSION

The results of the study allow us to state that buttermilk is suitable raw material for ice‐cream production. The substitution of milk with sweet or cultured buttermilk in ice‐cream formulation does not deteriorate the product quality. Ice cream from cultured buttermilk was characterized with higher acidity than control. Obtained products did not differ in over‐run value, but had different melting properties. The use of sweet buttermilk increased the stickiness of ice cream in comparison with control, what may indicate stabilizing effect of buttermilk. Buttermilk influenced color parameters of ice cream. Product from sweet buttermilk had the lowest a* and the highest b* value and product from cultured buttermilk had the highest lightness. Buttermilk ice cream, especially made from sweet buttermilk, had high assessed sensory properties, very similar to control ice cream made from milk. The use of buttermilk in ice‐cream formulation seems to be good alternative for this by‐product utilization and may be easily implemented in dairy industry. Beside good technological properties, the use of buttermilk may enhance nutritional value of frozen dairy desserts.

## 5 ETHICS STATEMENT

This study does not involve any human or animal testing.

## CONFLICT OF INTEREST

The authors declare that they do not have any conflict of interest.
